# Exploration of the Therapeutic Protocols and Underlying Molecular Mechanisms of Autologous Platelet‐Rich Plasma in the Treatment of Deep Second‐Degree Burns

**DOI:** 10.1111/jocd.70751

**Published:** 2026-02-25

**Authors:** Jian‐Guo Shi, Wei Liu, Hong‐Qiang Liu, Dan Rao, Jun‐Wei Huang, Yu‐Fei Zhao

**Affiliations:** ^1^ The Ninth People's Hospital of Chongqing Chongqing China; ^2^ The First People's Hospital of Chongqing High‐Tech Zone Chongqing China

**Keywords:** clinical application, deep second‐degree burn, growth factor concentration, immunohistochemistry, platelet‐rich plasma (PRP), standardization

## Abstract

**Background:**

Platelet‐rich plasma (PRP) has been widely applied in burn wound management due to its high concentration of bioactive growth factors. However, inconsistent clinical outcomes and the lack of standardized protocols regarding timing, frequency, and leukocyte content limit its broader clinical adoption.

**Aims:**

This study aimed to evaluate the therapeutic efficacy of PRP under different application protocols in patients with deep second‐degree burns and to explore the underlying molecular mechanisms, with the goal of informing standardized, evidence‐based PRP treatment strategies.

**Patients/Methods:**

Thirty‐three patients with deep second‐degree burns were enrolled and randomly assigned to five groups: a standard PRP group, a saline control group, an early intervention PRP group, a high‐frequency PRP group, and a leukocyte‐poor PRP group. Clinical outcomes included wound healing time, wound coverage rates at 2 and 3 weeks, bacterial culture positivity, and Vancouver Scar Scale scores at 3 months. Wound tissue and exudate samples were collected at predefined time points for immunohistochemical analysis of macrophage phenotypes (M1/M2) and quantification of growth factors (VEGF, PDGF, TGF‐β).

**Results:**

Compared with the control group, PRP treatment was associated with a shorter wound healing time, increased early wound coverage, reduced bacterial culture positivity, and improved scar outcomes. Early PRP intervention and the use of leukocyte‐poor PRP appeared to further enhance healing efficiency, which may be related to accelerated polarization of macrophages from the pro‐inflammatory M1 phenotype toward the anti‐inflammatory M2 phenotype. In contrast, high‐frequency PRP administration resulted in higher local growth factor levels but did not translate into additional clinical benefit; this lack of efficacy may be attributable to excessive expression of transforming growth factor‐β (TGF‐β), which could potentially counteract optimal tissue repair.

**Conclusions:**

PRP is an effective adjunctive therapy for small‐area deep second‐degree burns. Early application and leukocyte‐poor PRP appear optimal, whereas indiscriminately increasing treatment frequency is not recommended. Controlled modulation of PRP‐derived growth factors may be key to maximizing therapeutic benefit.

## 1 Introduction

According to data from the World Health Organization (WHO), approximately 11 million people suffer burn injuries each year, with around 180 000 burn‐related deaths globally [1]. Burns cause significant pain and trauma, and their incidence has been steadily increasing in recent years, particularly among the elderly and children [2]. The disruption of the skin barrier following a burn leads to a complex wound healing process, during which infection, pain, and scarring pose substantial burdens to patients [3, 4].

Platelet‐rich plasma (PRP) is a blood‐derived product characterized by a high concentration of platelets and is widely utilized in various fields of tissue regeneration due to its abundance of growth factors [5]. In recent years, PRP has been extensively applied to burn wounds and has been reported in numerous studies to exert significant effects in promoting wound healing, controlling infection, and reducing long‐term scarring [5, 6, 7, 8]. Although a substantial body of data on PRP has been reported and published, reproducible results remain limited [9]. Furthermore, there is a persistent lack of standardized protocols regarding PRP preparation, its clinical application in burn management, and its impact on therapeutic outcomes [10]. The efficacy of PRP in burn wound treatment remains inconsistent across studies, with significant variation among clinical centers in terms of preparation methods, timing of application, and wound selection criteria [11]. In their clinical case series, Karina et al. [12] administered PRP intervention between days 3 to 10 post‐burn and achieved favorable therapeutic outcomes; however, the rationale behind the timing and protocol design was not clearly explained. ArayaK et al. [13] in a murine model found that leukocyte‐poor PRP (LP‐PRP) demonstrated superior efficacy in controlling inflammation and pain in osteoarthritis. Nevertheless, experimental data on the application of LP‐PRP in burn models remain limited. Additionally, some clinical centers have reported no significant differences between PRP‐treated and control areas in terms of epithelialization, pain, adverse events, or infection rates in burn patients [14].

Based on this background, the present study aims to explore preliminary application strategies for platelet‐rich plasma (PRP) in the treatment of deep second‐degree burns by comparing its effects on infection control, wound healing time, and post‐burn scar formation under different intervention time points, application frequencies, and leukocyte contents. In addition, through the assessment of epithelialization, immunohistochemical changes, and local growth factor concentrations during the healing process, this study seeks to provide exploratory insights into the potential molecular mechanisms underlying the therapeutic effects of PRP, thereby offering a foundation for future studies aimed at establishing standardized PRP treatment protocols.

Notably, within the same overarching clinical trial framework, our research group has previously investigated the therapeutic efficacy of PRP across different platelet concentration gradients by comparing multiple concentration‐defined PRP groups, and the corresponding findings have been reported elsewhere. Building upon that work, the present study focuses on a complementary research question: by introducing additional experimental groups that vary in PRP intervention timing, application frequency, and leukocyte content, we further examine how different PRP application conditions influence clinical outcomes and biological responses in burn wound healing. Together, these studies aim to provide a more comprehensive and systematic understanding of PRP optimization in the management of deep second‐degree burns [15].

## 2 Materials and Methods

### 2.1 Patient Population

Patients hospitalized in the Department of Burns at our institution between August 2023 and March 2025 with a diagnosis of deep partial‐thickness (deep second‐degree) burns were prospectively screened for eligibility.

#### 2.1.1 Inclusion Criteria Were as Follows

1. Age between 18 and 55 years, irrespective of sex;
2. Burn injuries caused by flame, scalding, or steam, with electrical and chemical burns excluded;
3. Clinically confirmed deep partial‐thickness burns;
4. Admission within 48 h after injury;
5. Burn area involving 2%–7% of the total body surface area (TBSA);
6. Wounds that had received no prior intervention other than basic disinfection and temporary dressing outside the hospital.

#### 2.1.2 The Exclusion Criteria Included

7 Diagnosis of diabetes mellitus;
8 Severely contaminated or clinically infected wounds at presentation;
9 Burns involving the head, face, neck, or perineum;
10 Significant cardiac, hepatic, or renal dysfunction;
11 Hypoproteinemia (serum albumin < 30 g/L);
12 Thrombocytopenia (platelet count < 100 × 10^9^/L) or ongoing antiplatelet therapy;
13 Evidence of systemic infection, autoimmune disorders, or malignancy;
14 Current use of glucocorticoids or immunosuppressive agents;
15 Osteomyelitis or anaerobic bacterial infection;
16 Neurological or psychiatric disorders, or cognitive impairment affecting study compliance.

To eliminate the confounding effects of systemic inflammatory responses associated with extensive burns, we included only patients with deep second‐degree burns involving 2% to 7% of the total body surface area (TBSA). Additionally, patients with conditions that could potentially influence the efficacy of PRP therapy were excluded. Prior to the initiation of the study, all participants were informed in detail about the treatment procedures and provided written informed consent. Relevant ethical documentation is provided in the attachment.

### 2.2 Randomization, Blinding, and Experimental Subgroup Allocation

A simple randomization method was employed to allocate patients to treatment groups. Each participant was provided with five sealed envelopes labeled A, B, E, F, and G. Patients were asked to select one envelope, and the letter inside determined their assignment to a specific experimental subgroup and corresponding treatment regimen. This clinical trial was conducted under a double‐blind design, in which both patients and physicians performing outcome assessments were blinded to group allocation, while statisticians were unaware of the treatment assignments for individual participants.

Multiple experimental subgroups were established to evaluate the therapeutic performance of different PRP application strategies and to explore a potentially standardized PRP‐based treatment protocol for deep partial‐thickness burns. The subgroup employing a PRP regimen most commonly reported in the literature was designated as the standard PRP group, serving as the reference for intergroup comparisons [9, 12, 16]. Although PRP can be administered as liquid injections, gels, or scaffold‐based formulations, liquid PRP was selected in this study and injected subcutaneously along wound margins. This approach was chosen due to the technical complexity, higher cost, and limited quantitative control associated with gel and scaffold preparations.

### 2.3 Intervention Groups

A. Standard PRP Group.

Patients received PRP injections on post‐injury days 7 and 14. Platelet concentrations were maintained at 600–1000 × 10^9^/L (approximately fourfold baseline). PRP was injected subcutaneously along wound margins using a 30‐gauge needle, with 0.1 mL administered at 1–2 cm intervals.

B. Control Group.

Patients underwent identical procedures, except that sterile saline was administered instead of PRP.

E. Early‐Intervention PRP Group.

PRP injections were administered on post‐injury days 3 and 10, with platelet concentrations consistent with the standard group.

F. High‐Frequency PRP Group.

PRP injections were initiated on day 7 and repeated on days 10, 13, and 16, using the same platelet concentration range.

G. Leukocyte‐Poor PRP Group.

This group followed the standard PRP injection schedule, but PRP was prepared using gel‐separation tubes to substantially reduce leukocyte content while maintaining platelet concentrations within 600–1000 × 10^9^/L.

### 2.4 PRP Preparation and Quality Control

Baseline peripheral blood platelet counts were measured on each PRP administration day. The volume of whole blood collected was calculated based on an estimated 80% platelet recovery rate and a dosage of 1 mL PRP per 1% TBSA.

Whole blood samples anticoagulated with sodium citrate were subjected to a two‐step centrifugation protocol. After initial centrifugation at 1400 rpm for 10 min to separate erythrocytes, the supernatant was centrifuged at 2800 rpm for 10 min. A calculated volume of plasma was removed to obtain PRP with the target concentration. A 0.1 mL aliquot was retained for platelet and leukocyte quantification to ensure compliance with predefined standards.

For leukocyte‐poor PRP, separation gel tubes (Liuyang Yaxite Medical Technology Co. Ltd.; Registration No. Xiangxie Zhuzhun 20 152 220 024) were used according to manufacturer instructions.

### 2.5 Wound Care and Sample Collection

All wound management procedures were performed by physicians with more than 3 years of burn care experience. Dressing changes were conducted 2–3 times daily during the exudative phase and once daily during wound cleansing.

Wound care included cleansing with povidone‐iodine and normal saline, preservation of blister skin when feasible, gentle drainage of serous fluid, and meticulous debridement of necrotic tissue. Tangential excision was performed when thick eschar was present. After drying, nanosilver antimicrobial dressings (Shenzhen AGT Pharmaceutical Technology Co. Ltd.) were applied, followed by sterile gauze. All procedures adhered strictly to aseptic principles and international burn management guidelines [4]. Following complete epithelialization, patients were advised to wear compression garments or elastic bandages for 3–6 months.

### 2.6 Histopathology, Immunohistochemistry, and Growth Factor Analysis

On post‐injury days 5, 9, and 16, small full‐thickness tissue specimens were obtained from newly epithelialized wound margins, approximately 2 mm from the advancing epithelial edge, during routine dressing changes. Samples were processed for histological and immunohistochemical analysis. Additionally, on post‐injury days 5, 9, 11, 14, and 16, wound exudate samples from the central wound base were collected prior to disinfection during dressing changes and sent for bacterial culture.

Markers included **iNOS** and **CD206**, representing M1 and M2 macrophage phenotypes, respectively. Positive staining was identified by yellow–brown coloration under 400× magnification. Five randomly selected fields per section were analyzed to calculate mean positive cell counts [17].

Growth factor concentrations (VEGF, TGF‐β, PDGF) in wound exudate were quantified using ELISA kits (Registration No. Yuxie 20 202 400 032; NewScienTech Biotechnology Co. Ltd.) according to manufacturer protocols.

### 2.7 Outcome Measures

#### 2.7.1 Primary Outcome

Wound healing time, defined as the interval from injury to complete epithelialization, was independently assessed by two senior burn surgeons under blinded conditions [18].

#### 2.7.2 Secondary Outcomes

- Wound coverage rates at 2 and 3 weeks
- Bacterial culture positivity rates
- Scar severity assessed at 3 months using the Vancouver Scar Scale (VSS).

## 3 Statistical Analysis

The Kolmogorov–Smirnov (K‐S) test was used to assess the normality of measurement data (significance level *p* = 0.05), and Levene's test was used to examine the homogeneity of variance between groups (significance level *p* = 0.05). Data that met the assumptions of normal distribution and homogeneity of variance were expressed as “mean ± standard deviation.” One‐way analysis of variance (ANOVA) was employed for comparisons among multiple groups, and Dunnett's *t*‐test was used for post hoc pairwise comparisons using the standard experimental Group A as the reference. Categorical data were expressed as frequencies and percentages. Comparisons between two groups were conducted using the chi‐square test or continuity‐corrected chi‐square test, depending on the expected frequency. All statistical analyses were two‐tailed, with a significance level of α = 0.05. A *p* < 0.05 was considered statistically significant. Statistical analysis was performed using SPSS version 23.0.

## 4 Results

### 4.1 Patient Characteristics

A total of 33 patients with deep second‐degree burns were enrolled in this study, including 8 in Group A, 5 in Group B, 7 in Group E, 6 in Group F, and 7 in Group G. There were no statistically significant differences in baseline characteristics—including demographic data, height, weight, and burn area—among the groups, indicating good comparability between them. The platelet and leukocyte concentrations in the PRP preparations of each treatment group met the predefined inclusion criteria. as shown in (Table 1).

**TABLE 1:** Comparison of baseline data between different treatment groups.

Group	A (*n* = 8)	B (*n* = 5)	E (*n* = 7)	F (*n* = 6)	G (*n* = 7)
Sex (male/female)	5/3	3/2	5/2	4/2	5/2
Age (years)	36.63 ± 13.13	39.00 ± 9.75	40.29 ± 10.83	42.67 ± 12.71	40.14 ± 10.09
Weight (kg)	60.06 ± 9.73	62.30 ± 7.40	61.07 ± 9.32	60.08 ± 12.69	67.36 ± 11.58
Height (cm)	168.00 ± 10.37	167.00 ± 9.59	173.14 ± 8.07	172.50 ± 8.62	167.57 ± 10.01
Burn surface (%)	4.88 ± 1.73	4.80 ± 1.92	5.14 ± 2.27	3.67 ± 1.03	5.00 ± 2.00
Burn site(Limbs/Trunk)	7/1	4/1	7/0	5/1	6/1
Average platelets concentration (×109/L)	815.8 ± 82.3	0	796.4 ± 78.6	821.4 ± 73.2	803.4 ± 88.4
Average leucocytes concentration (×109/L)	161.5 ± 23.4	0	153.5 ± 19.4	149.5 ± 25.3	0.34 ± 0.18

*Note:* There were no statistically significant differences in baseline characteristics among the groups.

### 4.2 Wound Healing and Scarring

Compared with the standard experimental Group A, Group B exhibited a significantly longer wound healing time (*p* < 0.001) and a lower wound coverage rate at 2 weeks (*p* < 0.001). In contrast, Groups E and G showed significantly shorter healing times and higher wound coverage rates at 2 weeks (*p* < 0.05). At 3 months post‐healing, scar scores in Group B were significantly higher than those in Group A, while scores in Groups F and G were significantly lower (*p* < 0.05), as shown in (Table 2).

**TABLE 2:** Comparative analysis of wound healing and scar assessment scores among different treatment groups.

Group	A (*n* = 8)	B (*n* = 5)	E (*n* = 7)	F (*n* = 6)	G (*n* = 7)	F	Dunnett‐t
Wound healing time (day)	19.00 ± 2.73	26.20 ± 3.27	15.57 ± 2.23	19.17 ± 2.32	15.43 ± 2.64	15.47**	B > A**；A > E, G*
Wound coverage rate at weeks 2 (100%)	76.88 ± 11.00	41.00 ± 10.84	88.57 ± 9.00	77.50 ± 9.35	90.71 ± 7.87	23.42**	A > B**；E, G > A*
Wound coverage rate at weeks 3 (100%)	98.13 ± 3.72	80.00 ± 11.18	100.00 ± 0.00	98.33 ± 4.08	100.00 ± 0.00		
Scar assessment scores at 3 months post‐injury	7.38 ± 1.41	9.80 ± 1.48	6.71 ± 1.80	5.00 ± 1.41	5.14 ± 1.57	8.99**	B > A*；A > F, G*

*Note:* Dunnett's *t*‐test was performed using Group A as the reference group.

* *p* < 0.05.

** *p* < 0.001.

### 4.3 Bacterial Culture Results

Compared to Group A, Group B had a higher rate of positive bacterial cultures, while Groups E and G had significantly lower positive culture rates (*p* < 0.05). Notably, early PRP intervention (Group E) markedly reduced early‐stage bacterial detection rates, as presented in (Table 3).

**TABLE 3:** Comparison of bacterial culture results among different groups.

	Bacterial culture positivity rate (day 5)	Bacterial culture positivity rate (excluding day 5)	Control group	*X* ^2^
A (*n* = 8)	37.5% (3/8)	40.6% (13/32)	B	0.037
			E	0.009
			F	0.614
			G	0.024
B (*n* = 5)	40% (2/5)	70% (14/20)		
E (*n* = 7)	0% (0/7)	10.7% (3/28)		
F (*n* = 6)	33.3% (2/6)	46.9% (15/32)		
G (*n* = 7)	42.9% (3/7)	14.3% (4/28)		

*Note:* Culture results on days 9, 11, 14, and 16 were analyzed as independent samples. Bacterial culture positivity rate (excluding day 5) = Number of positive bacterial cultures (excluding day 5)/Total number of bacterial cultures performed (excluding day 5), i.e., 4 × number of patients per group.

### 4.4 Macrophage Quantification

**FIGURE 1:**
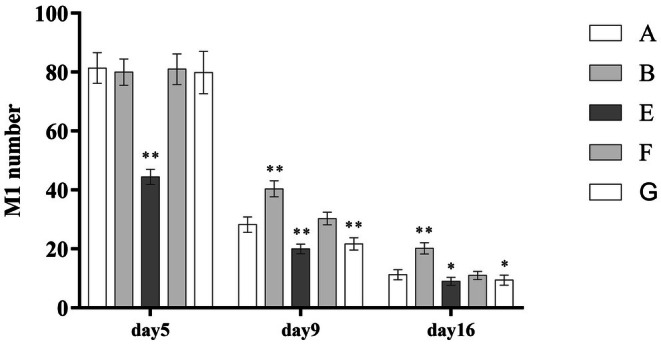
Comparison of macrophage M1 numbers.

**FIGURE 2:**
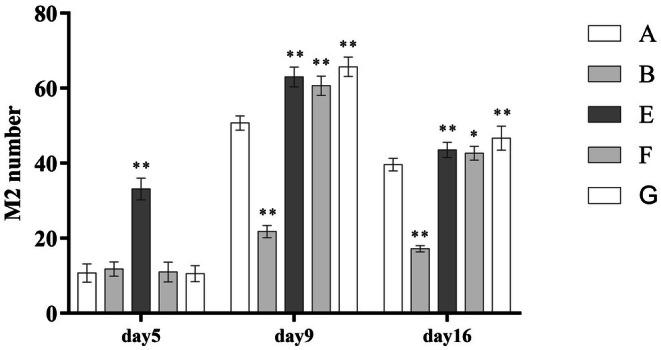
Comparison of macrophage M2 numbers.

During the process of burn wound healing, the number of M1 macrophages in wound tissue gradually decreased, while M2 macrophages increased. PRP treatment significantly accelerated this transition (Group B had more M1 and fewer M2 macrophages than Group A, with statistically significant differences). Early PRP intervention (Group E) and leukocyte‐poor PRP treatment (Group G) led to a faster M1‐to‐M2 macrophage transition (*p* < 0.001). In the high‐frequency PRP group (Group F), the number of M2 macrophages was significantly increased during wound healing, as shown in (Table 4) and (Figure 1, Figure 2).

**TABLE 4:** Comparison of M1 and M2 Macrophage Counts Across Different Groups.

Group	A(*n* = 8)	B(*n* = 5)	E(*n* = 7)	F(*n* = 6)	G(*n* = 7)	F	Dunnett‐t
Day 5 number of M1	81.38 ± 5.21	80.00 ± 4.47	44.43 ± 2.57	81.00 ± 5.22	79.86 ± 7.17	67.11**	A > E**
Day 9 number of M1	28.25 ± 2.60	40.40 ± 2.70	20.00 ± 1.63	30.33 ± 2.16	21.71 ± 2.06	74.81**	B > A**; A > E, G**
Day 16 number of M1	11.25 ± 1.67	20.20 ± 1.92	9.00 ± 1.41	11.00 ± 1.41	9.43 ± 1.72	42.84**	B > A**; A > E, G*
Day 5 number of M2	10.75 ± 2.43	11.80 ± 1.92	33.14 ± 2.91	11.00 ± 2.61	10.57 ± 2.15	112.69**	A < E**
Day 9 number of M2	50.75 ± 1.91	21.80 ± 1.64	63.00 ± 2.65	60.67 ± 2.58	65.71 ± 2.56	327.28**	B < A**; A < E, F, G**
Day 16 number of M2	39.63 ± 1.69	17.20 ± 0.84	43.57 ± 1.99	42.67 ± 1.86	46.71 ± 3.20	169.05**	B < A**; A < E, G**; A < F*

*Note:* Dunnett's *t*‐test was performed using Group A as the reference group.

* *p* < 0.05.

** *p* < 0.001.

### 4.5 Growth Factor Concentrations in Wound Exudate

**FIGURE 3:**
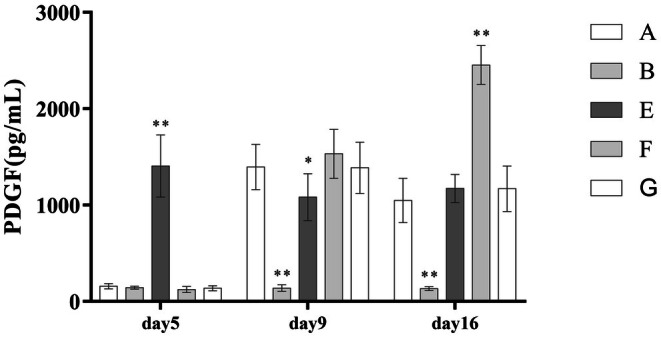
Comparison of PDGF concentrations.

**FIGURE 4:**
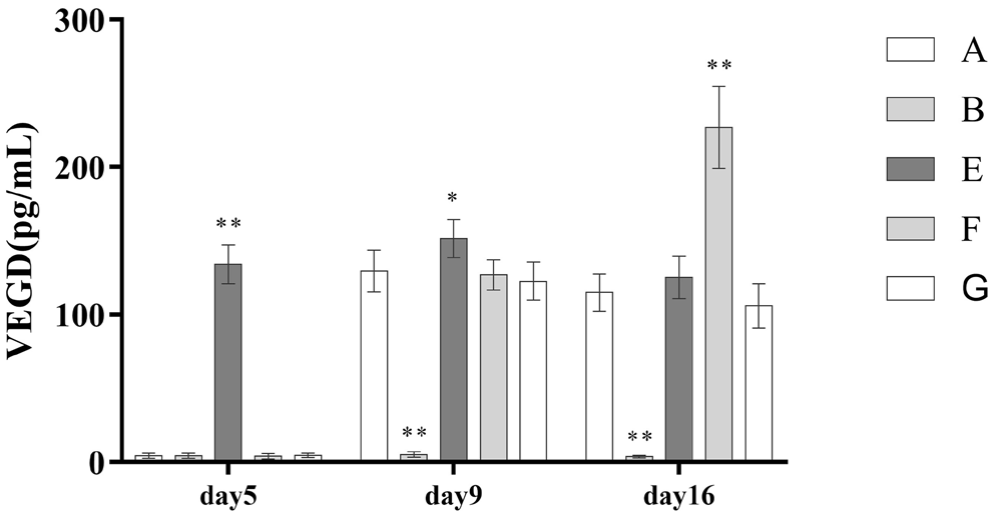
Comparison of VEGF concentrations.

**FIGURE 5:**
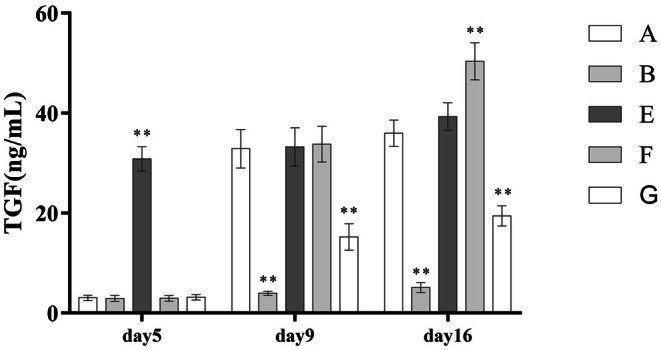
Comparison of TGF‐β concentrations.

Following PRP treatment, concentrations of VEGF, TGF‐β, and PDGF in wound exudate increased significantly. Compared with the standard PRP treatment group (Group A), the high‐frequency intervention group (Group F) showed even higher levels of all three growth factors. However, the leukocyte‐poor PRP group (Group G) demonstrated a significantly reduced concentration of TGF‐β. These differences were statistically significant (*p* < 0.001), as detailed in (Table 5) and (Figures 3, 4, 5).

**TABLE 5:** Comparison of Growth Factor Concentrations in Wound Exudates Across Different Groups.

Group	A(*n* = 8)	B(*n* = 5)	E(*n* = 7)	F(*n* = 6)	G(*n* = 7)	F	Dunnett‐t
TGF‐β5 (ng/mL)	3.06 ± 0.53	2.91 ± 0.60	30.84 ± 2.43	2.95 ± 0.57	3.17 ± 0.53		
TGF‐β9	32.88 ± 3.85	3.96 ± 0.42	33.23 ± 3.84	33.78 ± 3.58	15.23 ± 2.66	100.91**	A > B**; A > G**
TGF‐β16	35.97 ± 2.65	5.13 ± 1.01	39.31 ± 2.78	50.37 ± 3.69	19.45 ± 2.03	262.02**	A > B**; A > G**; F > A**
PDGF5 (pg/mL)	158.00 ± 27.30	143.60 ± 14.06	1406.43 ± 321.74	124.67 ± 30.78	138.29 ± 26.20		
PDGF9	1396.13 ± 236.13	139.80 ± 32.92	1082.57 ± 244.70	1532.50 ± 254.56	1387.57 ± 265.09	32.15**	A > B**; A > E*
PDGF16	1048.25 ± 230.10	134.20 ± 20.27	1173.00 ± 146.52	2454.33 ± 201.72	1170.43 ± 236.29	103.06**	A > B**; F > A**
VEGF5 (pg/mL)	4.38 ± 1.69	4.40 ± 1.67	134.14 ± 13.12	4.00 ± 1.79	4.57 ± 1.51		
VEGF9	129.50 ± 14.25	5.20 ± 1.92	151.71 ± 12.96	127.00 ± 10.04	122.71 ± 12.91	128.74**	A > B**; E > A*
VEGF16	115.00 ± 12.69	3.80 ± 0.84	125.29 ± 14.47	227.00 ± 27.84	106.00 ± 15.13	126.47**	A > B**; F > A**

*Note:* Dunnett's *t*‐test was performed using Group A as the reference group.

* *p* < 0.05.

** *p* < 0.001.

## 5 Discussion

The regenerative potential of PRP is primarily attributed to the abundant release of growth factors contained within platelets. These include platelet‐derived growth factor (PDGF), epidermal growth factor (EGF), vascular endothelial growth factor (VEGF), and transforming growth factor (TGF), among others [18]. These growth factors contribute to burn wound healing by promoting chemotaxis, cellular adhesion, mitosis, proliferation, and angiogenesis [19]. In a murine burn model, Bakinam M.H. Tammam [20] and colleagues found that following PRP treatment, VEGF levels in the burn area were significantly elevated, while pro‐inflammatory markers such as MMP‐9 were reduced, suggesting enhanced angiogenesis and epidermal regeneration. Neovascularization is critically important in post‐injury wound repair, and VEGF is one of the most potent mediators of angiogenesis, playing a key role in regulating vascular permeability and enhancing cell migration, spreading, and survival [21]。In our study, burn patients in Group A who received PRP treatment showed markedly elevated concentrations of VEGF, PDGF, and other growth factors in wound exudates compared to the control Group B. Histological sections also showed higher degrees of epithelialization, consistent with previous findings. Additionally, activated macrophages within the wound played a vital role in healing. Early in the wound healing process, iNOS‐expressing M1 macrophages predominate and secrete pro‐inflammatory cytokines, thereby amplifying the inflammatory response; however, excessive or prolonged inflammation may delay tissue repair. At later stages of healing, CD206‐positive M2 macrophages become increasingly dominant and contribute to wound repair by promoting endothelial cell differentiation, proliferation, and migration, as well as other regenerative processes [17]. In our study, the PRP‐treated Group A exhibited higher CD206 expression and lower iNOS expression compared with Group B, suggesting that PRP facilitates a shift in burn wounds from an M1‐dominant to an M2‐dominant macrophage profile, thereby promoting the wound healing process.

Currently, numerous animal experiments and clinical studies have been published regarding the use of PRP in the treatment of burn wounds. However, there remains significant variation among different centers in terms of PRP preparation methods, concentration selection, and timing of administration. These practices are often empirical, resulting in poor reproducibility across studies [6, 8, 22]. In our study, our center established a standardized PRP experimental group based on protocols derived from the majority of existing clinical research and implemented strict quality control to regulate platelet concentrations in PRP products, ensuring the reproducibility of the experiments. We designed multiple experimental subgroups to investigate various parameters, including different time points of PRP application, varying frequencies of administration, and the presence or absence of leukocyte enrichment. By comparing the therapeutic outcomes across these subgroups, we aimed to explore a standardized protocol for the application of PRP in the treatment of deep second‐degree burns.

On a temporal level, after burn injury, α‐granules within platelets release signaling molecules such as cytokines, chemokines, and microRNAs. These signals act to recruit leukocytes to the injury site before subsequently activating and modulating their function [23]. Once platelets have arrived, leukocytes release a large array of additional growth factors and signaling proteins, which are responsible for orchestrating the spatial and temporal regulation of cell proliferation and differentiation—an essential requirement for the wound healing process to transition from the hemostatic and inflammatory phases into the proliferative and remodeling stages [24]. This raises the question: can early intervention with PRP accelerate this progression, shorten the inflammatory phase, and expedite the onset of tissue proliferation and remodeling? Our experimental findings support this hypothesis. Compared with group A, patients in group E (early intervention) showed significantly shorter wound healing times and higher wound coverage rates at both 2 and 3 weeks. Furthermore, group E exhibited fewer M1‐type macrophages and more M2‐type macrophages than group A, which indirectly supports our inference. Additionally, the early intervention group had a lower rate of positive bacterial cultures. Studies have indicated that in the early phase of burn injuries, the most common sources of microbial contamination are resident skin flora, particularly Staphylococcus, Streptococcus, and methicillin‐resistant *Staphylococcus aureus* (MRSA) [4]. PRP has been shown to be effective—at varying degrees—against common wound pathogens including MRSA, MSSA, extended‐spectrum β‐lactamase ( *E. coli* ), *Klebsiella pneumoniae* , and *E. coli* [11]. It is possible that early control of inflammation is also a critical factor in accelerating wound healing. However, in our experiment, all treatment groups received standard wound care using povidone‐iodine and silver ion antimicrobial moist dressings, which may have masked some instances of bacterial infection. A larger‐scale study with more basic and less antimicrobial‐intensive dressing protocols might yield clearer and more definitive results.

Studies have shown that after platelet activation within PRP, there is a burst‐like release of growth factors within the first 24 h, followed by a gradual and sustained release that stabilizes around 72 h post‐activation [25]. In theory, high‐frequency PRP injections would better align with this release profile and thereby promote burn wound healing more effectively. However, in our study, the high‐frequency intervention group did not show a statistically significant difference in wound healing time or wound coverage compared to the standard treatment group. Interestingly, group F exhibited significantly higher levels of secreted growth factors than group A, and even showed a greater enrichment of M2‐type macrophages. We suspect that the lack of marked improvement in wound healing in group F may be attributed to excessively high levels of TGF‐β. TGF‐β is known to have a dual role in tissue repair: while it can suppress inflammation and promote fibroblast differentiation to support tissue regeneration [20]. an excessive concentration can lead to pathological fibrosis and excessive collagen deposition. This, in turn, may counteract the beneficial effects of other growth factors and even the pro‐healing functions of highly expressed M2 macrophages, ultimately impairing wound healing [26]. An optimal concentration of TGF‐β is critical for maintaining a balance between endothelial cell regeneration and apoptosis, making it essential for effective tissue repair and inflammation control [27]. Since merely increasing the frequency of PRP application and blindly elevating growth factor concentrations does not necessarily yield positive results, the use of heparinized scaffolds for the sustained release of PRP may offer a promising strategy. These scaffolds enable continuous and controlled release of growth factors, helping to maintain their concentration within an optimal therapeutic window. However, the high cost and current limitations in mass production remain significant obstacles to their broader clinical application [28].

Moreover, the role of leukocyte‐rich PRP (Lr‐PRP) versus leukocyte‐poor PRP (Lp‐PRP) in tissue healing remains a topic of ongoing debate in the academic community, with no consensus reached across clinical studies. Some researchers have reported that autologous Lp‐PRP is more effective than Lr‐PRP in repairing intervertebral disc degeneration (IDD) [29]. This may be because Lr‐PRP contains abundant pro‐inflammatory cytokines (such as TNF‐α and IL‐1β), which can counteract tissue repair processes and additionally promote catabolic activity. Conversely, other studies suggest that high leukocyte concentrations in PRP can suppress wound inflammation and reduce the risk of infection [30]. The conflicting results across these studies may stem from differences in tissue healing mechanisms across various diseases, as well as inconsistencies in PRP preparation methods. In the context of burn wounds, the skin's protective barrier is compromised, making infection a major concern during healing. Burns also provoke intense local inflammatory responses [24]. From this perspective, the abundant leukocytes in Lr‐PRP might theoretically help control burn‐related infections. However, our study found the opposite: patients treated with Lp‐PRP exhibited shorter wound healing times and higher wound coverage rates at both 2 and 3 weeks post‐treatment. Moreover, the incidence of positive bacterial cultures in Group G (Lp‐PRP) was lower than in other groups. This finding aligns with immunological studies in burn models, including a neutrophil‐depletion experiment in diabetic mice, which demonstrated that wound healing actually progressed faster in the absence of neutrophils [31]. We speculate this may be due to the impaired chemotaxis, phagocytic function, and bactericidal activity of neutrophils after prolonged infiltration [32]. When neutrophils become hyperactivated, their effectiveness in bacterial clearance may paradoxically decrease, potentially hindering wound repair and increasing susceptibility to infection [33]. In addition, leukocyte‐derived acidic hydrolases may exacerbate local inflammation and prolong the inflammatory phase of burn wound healing, further delaying recovery [11]. In our experiment, VEGF and PDGF levels in Group G were comparable to those in Group A, but Group G had fewer M1‐type macrophages and more M2‐type macrophages, which supports our conclusions. Notably, TGF‐β concentrations in Group G were significantly lower than in Group A—an observation corroborated by previous studies [34], likely due to the reduced leukocyte content resulting in diminished TGF‐β release. This may be a key factor contributing to the faster healing and reduced scarring observed in Group G.

Finally, scar prevention and management remain among the most critical aspects of burn treatment. However, previous studies on the efficacy of PRP in scar modulation have yielded conflicting results [10]. In our experiment, both the leukocyte‐poor PRP group (Group G) and the high‐frequency treatment group (Group F) showed more significant improvements in scar outcomes compared to the standard treatment group. We speculate that this may be related to the physiological mechanisms underlying scar formation. Factors such as oxidative stress can impair processes like vascularization, fibrosis, and tissue remodeling during wound healing. PRP has been shown to counteract burn‐induced oxidative stress in rat models by reducing inflammatory cytokines such as IL‐6 and TNF‐α while increasing the expression of antioxidant enzymes like superoxide dismutase, thereby modulating the oxidative and inflammatory microenvironment of the wound [20]. Chronic inflammation is one of the most critical risk factors for hypertrophic scar formation [24], which may explain why PRP can alleviate scar hypertrophy in burn patients—and why the leukocyte‐poor group experienced reduced scarring. Additionally, previous studies have shown that modulating the TGF‐β signaling pathway can suppress scar formation in various wound models [35]. In a mouse burn model, researchers found that PRP treatment significantly downregulated the protein expression of MMP‐3 and TGF‐β, thereby inhibiting scar hypertrophy [36], This effect was thought to be due to the activation of a TGF‐β1 negative feedback mechanism triggered by excessive exogenous TGF‐β1. However, an overabundance of TGF‐β is problematic: it can disrupt the balance between endothelial cell proliferation and apoptosis, leading to excessive fibrosis and collagen deposition, which negatively affects wound healing and increases the risk of hypertrophic scarring [26]. These findings suggest that the optimal concentration of TGF‐β may vary across different stages of burn tissue healing, and that the expression of various TGF‐β isoforms is likely to be crucial. Strategies such as incorporating TGF‐β slow‐release systems or adding TGF‐β1 neutralizing antibodies into PRP preparations may therefore provide promising avenues for future research [26]. The above discussion on the molecular mechanisms of PRP is inferred from previous studies as well as our experimental results. The actual mechanisms, however, are likely more complex and multifaceted. Our study provides only a theoretical consideration, and a more detailed and accurate understanding of these mechanisms requires further investigation.

This study has several limitations. First, to ensure ethical compliance and effective wound management in the control group, silver‐based dressings were used instead of conventional gauze. However, potential interactions between silver ions and PRP‐derived growth factors cannot be excluded, which may act as a confounding factor influencing wound‐healing outcomes. Second, this was a single‐center study with a limited sample size, which restricts statistical power and generalizability. Although our findings suggest potential advantages related to PRP injection timing, treatment frequency, and leukocyte content, these observations should be interpreted as exploratory. Larger, multicenter clinical trials are required to validate these findings and to establish reliable, standardized PRP treatment strategies for burn wound management.

## 6 Conclusion

Our study demonstrates that the application of PRP in patients with small‐area deep partial‐thickness burns can significantly shorten wound healing time, reduce the risk of bacterial infection, and effectively alleviate subsequent scar hypertrophy. Regarding treatment strategies, we recommend early intervention with PRP and the use of leukocyte‐poor PRP preparations. However, indiscriminately increasing the frequency of PRP application is not advisable. In future research, integrating PRP with other biomaterials to enable the sustained release of growth factors—keeping their concentrations within an optimal range—may be key to facilitating the broader clinical application of PRP.

## Author Contributions

Jian‐Guo Shi and Wei Liu performed the study and wrote the paper. Wei Liu, Dan Rao, and Jun‐Wei Huang collected and analyzed the data, and made the tables and figures. Dan Rao and Hong‐Qiang Liu designed the study, edited the manuscript, and offered suggestions for this study. Jian‐Guo Shi and Wei Liu contributed equally to this work. Hong‐Qiang Liu and Dan Rao are co‐corresponding authors. Yu‐Fei Zhao was responsible for the manuscript submission.

## Funding

This work was supported by Natural Science Foundation of Chongqing Municipality (CSTB20233NSCQ‐MSX1062).

## Ethics Statement

The authors confirm that the ethical policies of the journal have been adhered to and the appropriate ethical review committee approval has been received. This study was reviewed and approved by the Medical Ethics Committee of Chongqing Ninth People's Hospital, with the ethical approval number Ref. No.: 2025‐KY‐(Ethics)‐015. Relevant ethical documentation is provided in the attached files.

## Conflicts of Interest

The authors declare that this clinical research was conducted with full academic independence. No financial or personal relationships that could inappropriately influence, or be perceived to influence, the work reported in this manuscript exist. The study was carried out solely for scientific and clinical purposes, without external sponsorship or conflicts of interest.

## Data Availability

The data that support the findings of this study are available from the corresponding author upon reasonable request.
